# Advanced Sensors for Intelligent Robotic Systems: Vision, Touch, and Dexterous Manipulation

**DOI:** 10.3390/s26092626

**Published:** 2026-04-23

**Authors:** Yanyan Wu, Xueqian Wang, Yajing Shen, Chongkun Xia

**Affiliations:** 1 School of Advanced Manufacturing, Sun Yat-Sen University, Shenzhen 518005, China; wuyy363@mail2.sysu.edu.cn; 2 Shenzhen Graduate School, Tsinghua University, Shenzhen 518055, China; wang.xq@sz.tsinghua.edu.cn; 3 Department of Electronic and Computer Engineering, Hong Kong University of Science and Technology, Hong Kong; eeyajing@ust.hk

## 1. Introduction and Current Trends in the Field

Advanced sensing is rapidly reshaping intelligent robotic systems by enabling robots to perceive, reason, and interact with the physical world under real-world uncertainty. Vision sensors provide rich spatial and semantic information, while tactile and force sensing deliver interaction cues that are fundamentally inaccessible to purely optical systems—such as contact state, incipient slip, frictional constraints, and distributed load transfer [1,2]. With the recent progress of AI-driven multimodal learning, perception is no longer an isolated front-end module; instead, it increasingly becomes a co-designed component tightly coupled with control and decision-making, closing the perception–action loop for robust grasping and dexterous manipulation [3,4].

Several trends are particularly evident. First, sensing solutions are moving toward task-oriented co-design, where hardware structures, sensor layouts, and decoding models are jointly optimized for manipulability and cost-effectiveness [5,6]. Second, there is increasing emphasis on robust perception in challenging environments, including low light, occlusions, dynamic disturbances, and long-term deployment constraints [7,8]. Third, fusion and compensation across modalities such as vision and touch, or proprioception and exteroception, as well as compensation for error sources such as zero drift, creep, and parameter uncertainty, are becoming essential for reliable closed-loop interaction [9]. Finally, the community continues to pursue deployable systems: low-cost, real-time, and engineering-validated solutions that can translate from lab prototypes to operational platforms in industrial automation, healthcare, and field robotics [10].

This Special Issue, “Advanced Sensors for Intelligent Robotic Systems: Vision, Touch, and Dexterous Manipulation”, was organized to gather innovative sensing technologies and perception–control methodologies that bridge the perception–action gap, with particular interest in vision–touch frameworks, sensor fusion, and practical validation in manipulation and robotics applications.

## 2. Scope of Special Issue and Contributions

This Special Issue collates 11 accepted papers (10 Articles and 1 Review) after a rigorous peer-review process. The accepted contributions span sensing hardware, perception algorithms, and closed-loop robotic systems, with representative topics including:

- tactile/force sensing and interaction intelligence for manipulation;
- robust vision-based perception (pose estimation, tracking, low-light sensing, and image enhancement);
- sensor-informed modeling, calibration, and control for complex robotic platforms;
- system-level integration and validation in healthcare, industrial, aerial, marine, and space robotics.

These contributions are outlined in more detail below.

In contribution 1, Wu et al. present a sparse strain-node tactile interface device that achieves high-resolution contact localization and three-axis force estimation with a small number of readout channels, combining structural design with learning-based decoding and sim-to-real calibration to improve accuracy while maintaining low hardware cost.

In contribution 2, Wen et al. propose a universal tool-end interaction force estimation approach for robotic tool manipulation. By integrating online-identifiable Newton–Euler tool dynamics, zero-drift identification, and an SNN-based compensation mechanism for wrist sensor creep, the method enables precise force/torque estimation across tool changes and grasp posture variations, supporting compliant manipulation control.

In contribution 3, Shan et al. develop a low-cost, real-time pipeline for multi-robot 6D pose estimation and multi-target tracking using a single RGB-D camera. Their approach enhances robustness under partial occlusion and visually similar targets via confidence filtering and tracking association strategies, enabling deployable state perception for multi-robot coordination.

In contribution 4, Ding et al. address vision sensing degradation under insufficient and uneven illumination by introducing a low-light waste-rock detection method that combines illumination-adaptive enhancement and detection-oriented feature modulation. This work demonstrates how perception models can be redesigned to improve reliability of sensor-based detection in harsh industrial environments.

In contribution 5, Liu et al. provide a comprehensive review of deep learning approaches for single-image super-resolution (SISR). By organizing methods around core components (datasets, upsampling strategies, objectives, and quality assessment), the review offers an accessible knowledge framework and highlights open challenges relevant to robotics perception, where resolution constraints frequently limit downstream recognition and tracking performance.

In contribution 6, Xue et al. report a cable-driven hybrid robot with series–parallel coupling, and develop modeling, workspace analysis, and self-calibration strategies for high-precision operation in large workspaces. The work emphasizes how multi-sensor system design and calibration pipelines underpin robust manipulation and automated grasping in complex hybrid robots.

In contribution 7, Hu et al. study kinematic modeling for cable-driven parallel robots with adaptive pulley kinematics, where pulley motion significantly affects cable length variation. By establishing an improved kinematics model and proposing a hybrid Levenberg–Marquardt and genetic algorithm solver, this work advances accurate modeling and high-precision kinematic solutions for CDPR systems.

In contribution 8, Guerreiro et al. present an automatic grasping system for multi-drone parcel delivery, combining a grasping mechanism, marker-based pose estimation, and a hybrid MPC strategy that coordinates aerial motion and gripper actuation across discrete phases. This paper highlights the role of perception-guided, sensor-informed control in autonomous aerial manipulation.

In contribution 9, Ompico et al. develop a robot-assisted TMS localization system that integrates a 3D camera with dual capacitive sensors for coil tilt detection and contact feedback. The proposed system emphasizes cost-effective sensing and human-interaction safety, illustrating how sensor fusion can enhance reproducibility and reduce operator dependency in clinical robotic assistance.

In contribution 10, Hu et al. investigate base pose stabilization for a post-capture space robot–satellite combined system. By integrating dynamic feedforward compensation with PD control and validating via simulation under disturbance and uncertainty, this contribution demonstrates sensor-informed modeling and control for robust operation in space robotic missions.

In contribution 11, Liu et al. present a real-time deep-sea mooring system that integrates inductive telemetry with satellite communication and multi-sensor arrays for long-term deployment. While outside conventional manipulation settings, this work exemplifies robust multi-sensor integration, reliability engineering, and real-world validation—capabilities increasingly essential for field robotics sensing infrastructures.

## 3. Conclusions

As Guest Editors, we are pleased with the outcome of this Special Issue and the breadth of sensing innovations and system-level validations represented by the accepted papers. Collectively, these contributions illustrate a clear direction for intelligent robotics: sensing solutions must be accurate, robust, and deployable, while being designed together with learning and control to close the perception–action loop in contact-rich tasks. We sincerely thank all authors for their high-quality submissions and the reviewers for their careful evaluations and constructive suggestions. We also acknowledge the Sensors Editorial Office and managing team for their consistent support throughout the preparation, submission, and review processes.
